# The Navigation Guide—Evidence-Based Medicine Meets Environmental Health: Integration of Animal and Human Evidence for PFOA Effects on Fetal Growth

**DOI:** 10.1289/ehp.1307923

**Published:** 2014-06-25

**Authors:** Juleen Lam, Erica Koustas, Patrice Sutton, Paula I. Johnson, Dylan S. Atchley, Saunak Sen, Karen A. Robinson, Daniel A. Axelrad, Tracey J. Woodruff

**Affiliations:** 1 Oak Ridge Institute for Science and Education (ORISE) Postdoctoral Fellow, National Center for Environmental Economics, Office of Policy, U.S. Environmental Protection Agency, Washington, DC, USA; 2 Program on Reproductive Health and the Environment, University of California, San Francisco, Oakland, California, USA; 3 Department of Epidemiology and Biostatistics, University of California, San Francisco, San Francisco, California, USA; 4 Department of Health Policy & Management,; 5 Department of Medicine, and; 6 Department of Epidemiology, Johns Hopkins University, Baltimore, Maryland, USA; 7 National Center for Environmental Economics, Office of Policy, U.S. Environmental Protection Agency, Washington, DC, USA

## Abstract

Background: The Navigation Guide is a novel systematic review method to synthesize scientific evidence and reach strength of evidence conclusions for environmental health decision making.

Objective: Our aim was to integrate scientific findings from human and nonhuman studies to determine the overall strength of evidence for the question “Does developmental exposure to perfluorooctanoic acid (PFOA) affect fetal growth in humans?”

Methods: We developed and applied prespecified criteria to systematically and transparently *a*) rate the quality of the scientific evidence as “high,” “moderate,” or “low”; *b*) rate the strength of the human and nonhuman evidence separately as “sufficient,” “limited,” “moderate,” or “evidence of lack of toxicity”; and *c*) integrate the strength of the human and nonhuman evidence ratings into a strength of the evidence conclusion.

Results: We identified 18 epidemiology studies and 21 animal toxicology studies relevant to our study question. We rated both the human and nonhuman mammalian evidence as “moderate” quality and “sufficient” strength. Integration of these evidence ratings produced a final strength of evidence rating in which review authors concluded that PFOA is “known to be toxic” to human reproduction and development based on sufficient evidence of decreased fetal growth in both human and nonhuman mammalian species.

Conclusion: We concluded that developmental exposure to PFOA adversely affects human health based on sufficient evidence of decreased fetal growth in both human and nonhuman mammalian species. The results of this case study demonstrate the application of a systematic and transparent methodology, via the Navigation Guide, for reaching strength of evidence conclusions in environmental health.

Citation: Lam J, Koustas E, Sutton P, Johnson PI, Atchley DS, Sen S, Robinson KA, Axelrad DA, Woodruff TJ. 2014. The Navigation Guide—evidence-based medicine meets environmental health: integration of animal and human evidence for PFOA effects on fetal growth. Environ Health Perspect 122:1040–1051; http://dx.doi.org/10.1289/ehp.1307923

## Introduction

Evidence-based decision making in environmental health requires synthesizing research from human and nonhuman (i.e., animal) evidence to reach overall strength of evidence conclusions, and is an integral part of hazard identification and risk assessment [National Research Council (NRC) 2009]. However, numerous shortcomings of current methods for research synthesis in environmental health have been identified, indicating, in particular, that a robust, systematic, and transparent methodology is needed (NRC 2011). To the extent that science informs decision making, limitations in the methods for evaluating the strength of evidence in environmental health impedes our capability to act on the science in a timely way to improve health outcomes (Woodruff and Sutton 2014).

In the clinical sciences, methods of research synthesis—which integrate transparent and systematic approaches to evidence collection and evaluation—have been developed and refined over the past three decades and have played a transformative role in evidence-based decision making for medical interventions (GRADE Working Group 2012; Higgins and Green 2011). For example, a systematic review and cumulative meta-analysis (continually updating the meta-analysis with results from more recent clinical trials) in cardiovascular medicine found discrepancies between recommendations by clinical experts and meta-analytic evidence. Experts often did not recommend treatments that pooled evidence demonstrated as effective, or they recommended treatments shown to have no effect or to be potentially harmful (Antman et al. 1992). As a result, systematic and transparent methods of research synthesis are now relied upon in clinical medicine to determine which interventions should be offered to patients. Empirical evidence shows that this approach to evidence-based medicine is superior compared with traditional expert-based narrative reviews (Antman et al. 1992; Fox 2010; Rennie and Chalmers 2009).

However, methods of research synthesis used in the clinical sciences are not fully applicable to environmental health, primarily because of the differences in evidence streams and decision contexts between the two (Woodruff et al. 2011a). In particular, robust methods for evaluating nonhuman evidence streams and fully developed methods for evaluating observational human studies are lacking (Woodruff et al. 2011a). In response to the need for improved methods of research synthesis in environmental health, beginning in 2009, an interdisciplinary collaboration of 22 clinicians and scientists from federal and state government agencies, academic institutions, and nongovernmental organizations developed the Navigation Guide systematic review method (see Supplemental Material, “Navigation Guide Workgroup Members,” for additional details) (Woodruff et al. 2011a). The Navigation Guide methodology incorporates best practices in research synthesis from clinical and environmental health science and provides an approach for evaluating and integrating human and nonhuman evidence streams (Woodruff et al. 2011a). The result of applying the Navigation Guide methodology is a concise statement about the quality and strength of the body of evidence of a contaminant’s toxicity.

We undertook a case study to apply the Navigation Guide methodology and demonstrate the applicability of systematic and transparent methods of research synthesis to environmental health. In two systematic reviews we assessed human and nonhuman scientific evidence, including rating the quality and strength of evidence (Johnson et al. 2014 and Koustas et al. 2014, respectively). In the present review, we integrated the strength of the human and nonhuman evidence ratings from these papers into an overall strength of evidence rating for an association between exposure to perfluorooctanoic acid (PFOA) and fetal growth. We selected this question because *a*) PFOA has been in widespread use for > 50 years [Prevedouros et al. 2006; U.S. Environmental Protection Agency (EPA) 2012]; *b*) PFOA is ubiquitous in the blood of the general U.S. population, including pregnant women, women of child-bearing age, and in cord blood (Apelberg et al. 2007; Mondal et al. 2012; Woodruff et al. 2011b); *c*) fetal growth is a health outcome of great public health importance (Institute of Medicine 2007); and *d*) we were aware of multiple epidemiological and mammalian toxicological studies addressing this question available in the peer-reviewed scientific literature.

## Methods

The Navigation Guide outlines four steps, the first three of which were addressed in this case study. We assembled a review team to include experts in the fields of risk assessment, environmental health, epidemiology, biology, systematic review, and toxicology to develop a protocol to address each step: *1*) Specify the study question; *2*) select the evidence; and *3*) rate the quality and strength of the evidence (Woodruff et al. 2011a). The methods for each step were outlined beforehand in protocols developed separately for human and nonhuman evidence [University of California, San Francisco (UCSF) Program on Reproductive Health and the Environment 2013]. The fourth and final step of the Navigation Guide—that is, grade the strength of the recommendation (to determine the final recommendation for public health protection)—was not addressed in this case study due to resource constraints. Additional information regarding the Navigation Guide methodology and the review team is available elsewhere (Woodruff and Sutton 2014).

Steps 1–3 are briefly summarized below for the human and nonhuman evidence streams. The detailed methods for each step in the human and nonhuman evidence streams are presented in separate papers (Johnson et al. 2014; Koustas et al. 2014). Here we describe a novel feature of the Navigation Guide systematic review method: the process of integrating the quality and strength of the human and nonhuman bodies of evidence into a final strength of evidence conclusion about human toxicity.

### Step 1. Specify the Study Question

Our overall objective was to integrate scientific findings from human and nonhuman studies to rate the strength of evidence for the question “Does developmental exposure to PFOA or its salts affect fetal growth in humans?” We used a PECO framework (population, exposure, comparator, and outcomes) to develop our question (Higgins and Green 2011). We established two separate PECO statements, one for human and one for nonhuman evidence (Table 1). These PECO statements were used to develop the search terms and inclusion/exclusion criteria for our systematic review in the next step.

**Table 1:** Human and animal PECO (population, exposure, comparator, outcome) statements.

PECO element	Human evidence	Animal evidence
Study question	Does developmental exposure to PFOA affect fetal growth in humans?	Does developmental exposure to PFOA affect fetal growth in animals?
Participants	Humans that are studied during the reproductive/developmental time period (before and/or during pregnancy or development)	Animals from non­human species that are studied during the reproductive/developmental time period (before and/or during pregnancy for females or during development for embryos)
Exposure	Exposure to PFOA (CAS# 335-67-1) or its salts during the time before pregnancy and/or during pregnancy for females or directly to fetuses	One or more oral, subcutaneous, or other treatment(s) of any dosage of PFOA (CAS# 335-67-1) or its salts during the time before pregnancy and/or during pregnancy for females or directly to embryos
Comparators	Humans exposed to lower levels of PFOA than the more highly exposed humans	Experimental animals receiving different doses of PFOA or vehicle-only treatment
Outcomes	Effects on fetal growth, birth weight, and/or other measures of size, such as length	Changes in fetal weight near term (e.g., embryonic day 18 for mice, embryonic day 21 for rats), birth weight, and/or other measures of size at term or birth, such as length

### Step 2. Select the Evidence

We implemented a comprehensive search strategy to identify human and nonhuman studies from the scientific literature. We searched a variety of databases to identify studies, using search terms tailored for each database based on our PECO statements. We also hand searched the reference lists of included articles to identify additional studies. Our search was not limited by language or publication date.

All results were screened using prespecified selection criteria and a structured form in DistillerSR (Evidence Partners; http://www.systematic-review.net). Studies were excluded if one or more of the following criteria were met:

The article did not include original data (i.e., a review article)
The study did not evaluate humans or animals (i.e., *in vitro* studies)
The study subjects were not exposed to PFOA or exposure was not during the reproductive or developmental time period;
Fetal growth or birth weight was not measured.

From eligible studies, we collected details of the study characteristics, exposure assessment, outcome measurements, and other information used to assess risk of bias using either a structured form in DistillerSR or a Microsoft Access 2007 database. We contacted study authors to request any data needed for the analysis that were not reported in the published articles.

*Statistical analysis.* For both human and nonhuman studies, we assessed study characteristics (i.e., study features and biological heterogeneity) to identify studies suitable for meta-analysis.

We used a random-effects meta-analysis approach using the DerSimonian-Laird estimator of potential statistical heterogeneity across studies (DerSimonian and Laird 1986). All computations for the human studies meta-analysis were done in STATA, version 12.1 (StataCorp LP) using the “metaan” command. All computations for the meta-analysis of nonhuman studies were performed in the programming environment R, version 2.13.1 (R Development Core Team; http://www.R-project.org/), using the package “metafor” (Viechtbauer 2010).

To visually assess the possibility of publication bias in a meta-analysis, we considered producing a funnel plot of the estimated effects. However, tests for funnel plot asymmetry are not recommended when there are fewer than 10 studies because test power is usually too low to distinguish chance from real asymmetry (Sterne et al. 2011). Because our meta-analysis for animals and humans was limited to < 10 studies each, we did not produce a funnel plot.

*Statistical heterogeneity.* We tested study variability using Cochran’s *Q* statistic to detect whether differences in the estimated effect between studies could be explained by chance alone or due to nonrandom sources of variability between studies. We considered a *p*-value < 0.05 to be statistically significant. We also calculated the *I*^2^ statistic, which estimates the percentage of variation across studies due to heterogeneity rather than chance (Higgins et al. 2003). To assess the impact of existing study heterogeneity on the meta-analysis, we considered the magnitude/direction of effect estimates, the Cochrane Collaboration’s guidelines to interpreting the *I*^2^ values (Deeks et al. 2011), and statistical tests of heterogeneity (e.g., by assessing the *p*-value from the Cochran’s *Q* test).

*Sensitivity analysis.* We conducted sensitivity analyses to investigate the effect on meta-analysis results. For the human evidence stream, we explored the effect of removing one included data set at a time, as well as adding back in an excluded study. For the nonhuman evidence stream, we explored the effect of removing one included data set at a time.

### Step 3. Rate the Quality and Strength of the Evidence

**Figure 1:**
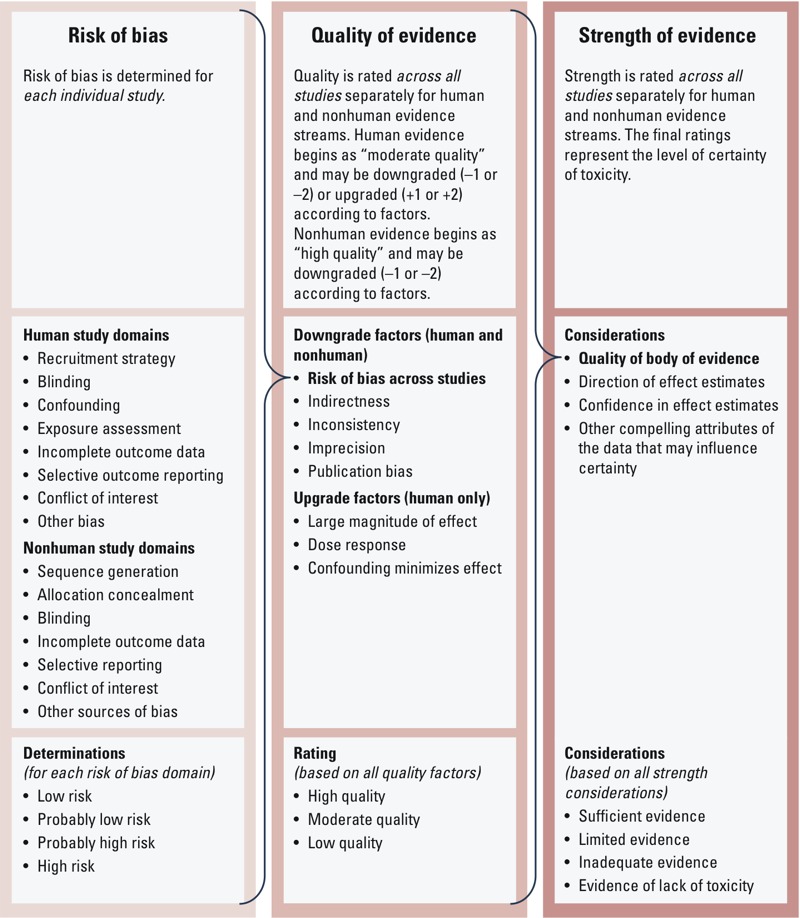
Overview of the Navigation Guide systematic review process to rate the quality and strength of the evidence.

Figure 1 provides an overview of the rating process and includes risk of bias domains, quality of evidence factors, and strength of evidence considerations used to rate the quality and strength of the human and nonhuman evidence. We used this rating process to evaluate the human and nonhuman evidence streams separately.

*Risk of bias across studies.* Two review authors (J.L. and E.K. for nonhuman studies, and P.I.J. and D.S.A. for human studies) independently assessed each included study for the risk of bias, defined as study characteristics that may introduce a systematic error in the magnitude or direction of the results (Higgins and Green 2011). We developed an instrument for rating risk of bias by modifying existing risk of bias instruments used in human studies in the clinical sciences, that is the Cochrane Collaboration’s risk of bias tool (Higgins and Green 2011) and the Agency for Healthcare Research and Quality’s (AHRQ) criteria (Viswanathan et al. 2012).

The Cochrane Collaboration’s risk of bias tool does not currently include a specific domain for bias related to study funding source, but this is an area of active discussion among its members (Bero 2013; Sterne 2013). The Cochrane Collaboration has recognized the importance of identifying study funding source, which has been empirically shown to be associated with biases (Krauth et al. 2014; Lundh et al. 2012). However, there is currently limited consensus on whether the study funding source should be included as a separate risk of bias domain or generally reported and commented on within the Cochrane systematic review (Bero 2013; Sterne 2013). A recent report from the NRC recommended that the U.S. EPA consider funding sources in their risk of bias assessment conducted for systematic reviews (NRC 2014).

On the basis of the recommendations mentioned above, we also included study funding source and declared financial conflicts of interest as a potential source of bias (i.e., whether the study received support from a company, study author, or other entity having a financial interest in the outcome of the study). We refer to this risk of bias domain generally as “conflicts of interest,” although for this particular case study we assessed only competing financial interests within this domain. A complete list of the human and nonhuman risk of bias domains is presented in Figure 1; detailed descriptions of each domain are provided by Koustas et al. (2014) and Johnson et al. (2014). Each risk of bias domain was assigned a determination of “high,” “probably high,” “low,” or “probably low” risk of bias based on previously determined criteria (Johnson et al. 2014; Koustas et al. 2014). We followed the GRADE (Grading of Recommendations Assessment, Development and Evaluation) principles for evaluating overall risk of bias by judiciously considering the frequency of each type of bias across all studies, evaluating the extent to which each study contributed toward the magnitude of effect estimate, and being conservative in the judgment of rating down (i.e., evidence was rated down only if risk of bias was clearly a substantial issue across most studies) (Viswanathan et al. 2012).

*Rating the quality of evidence across studies.* Each of the review authors compared the results of the systematic review to the Navigation Guide factors and considerations for rating the quality of the evidence as a way to initiate the group discussion and gather all perspectives for consideration. The Navigation Guide rating method (Woodruff et al. 2011a) was applied according to explicit written directions (Johnson et al. 2014; Koustas et al. 2014). The possible ratings for the overall quality of evidence were “high,” “moderate,” or “low.” Adapting the GRADE method as guidance, we first assigned a prespecified initial quality rating to the body of evidence, and then considered adjustments (downgrades or upgrades) to the quality rating based on the characteristics of the studies constituting the body of evidence to arrive at a final rating determination (Balshem et al. 2011).

We assigned prespecified initial ratings of “moderate” for the body of human observational data and “high” for the experimental nonhuman data, independent of the specifics of included studies; these characteristics were then evaluated later for upgrading or downgrading this rating as appropriate. Our rationale to assign the initial rating of “moderate” was based on the absolute and relative merit of human observational data in evidence-based decision making in environmental and clinical sciences. Human observational studies generally are recognized as being a reliable source of evidence in the clinical sciences and the preferred method for evaluating disease etiology (Institute of Medicine et al. 2008). Because ethical considerations virtually preclude experimental human data from the environmental health evidence stream, human observational studies are typically the “gold standard” of this evidence base. In comparison, randomized animal experiments have a high level of study design control (including level and duration of exposure) and test a study population of limited heterogeneity (inbred strains of laboratory animals). Thus, these data were the most comparable to human randomized controlled trials (RCTs) in the clinical sciences. To reflect this comparability, we assigned the experimental nonhuman data (both mammalian and nonmammalian) the initial rating of “high” as appropriate.

We assessed the overall body of human evidence for downgrading and upgrading the prespecified “moderate” quality rating based on eight factors—five for downgrading and three for upgrading. Our criteria for evaluating evidence from studies incorporate elements similar to the Bradford Hill considerations (i.e., consistency of effect, strength of effect, biologic gradient, incorporating experimental evidence from animal studies) and elements from other frameworks for evaluating scientific evidence [the U.S. Preventive Services Task Force (Sawaya et al. 2007) and the International Agency for Research on Cancer (IARC) 2006].

We decided to evaluate the nonhuman evidence separately for mammalian versus nonmammalian evidence because of fundamental biological differences between the two and because of the lower quality (i.e., high risk of bias) of the nonmammalian evidence. We evaluated each using the same five factors for downgrading the prespecified “high” quality rating, but did not consider any upgrades to the quality rating because the initial rating was already set at “high.” Consistent with GRADE guidelines (Guyatt et al. 2011d), we did not upgrade or downgrade the body of evidence unless there was a compelling rationale to do so.

Each of the nine review authors applied their expert judgment to review the bodies of evidence and independently graded the quality of evidence based on the presence of these factors using detailed instructions. The instructions to review authors contained specific information on how to evaluate the quality of evidence [see Supplemental Material, “Instructions for rating the quality and strength of human and nonhuman evidence” (UCSF Program on Reproductive Health and the Environment 2013)]. Possible ratings were 0 (no change), –1 (one-level downgrade), or –2 (two-level downgrade). Each overall body of evidence was evaluated for downgrading based on consideration of five factors:

Risk of bias across studies: Evidence streams were rated down if most of the relevant evidence came from studies that had high risk of bias, although review authors were instructed to be conservative in the judgment of rating down. In other words, review authors were instructed to rate down only if they judged that there was a substantial risk of bias in the body of available evidence. Furthermore, review authors were instructed not to assess factors by averaging across studies (e.g., if some studies had “low” risk of bias, a similar number of studies had “probably high” risk of bias, and a similar number of studies had “high” risk of bias, the quality should not be downgraded solely by averaging the risk of bias ratings).
Indirectness: Following GRADE guidelines (Guyatt et al. 2011a), evidence streams were rated down if substantial differences existed between the study population, exposure, comparator, or outcome measured relevant to our study question. Potential sources of indirectness included a study population or intervention/exposure that was so different from that of interest that there was a compelling reason to think that the magnitude of effect would differ substantially, or studies that reported on surrogate end points instead of the outcome of interest. In contrast to GRADE, our prespecified assumption was that animal evidence provides direct evidence for human health. However, in applying GRADE principles to the Navigation Guide, animal evidence will be rated down if it is determined to be a biologically inappropriate nonhuman model for the health outcome under study.
Inconsistency: Evidence streams were rated down if studies conducted in similar human populations or animal species had widely different estimates of effect (unexplained heterogeneity or variability in results). The following considerations were used to indicate potential inconsistency: *a*) point estimates varied widely across studies; *b*) confidence intervals (CIs) showed minimal or no overlap for similar studies of comparable size; *c*) the statistical test for heterogeneity had a low *p*-value (*p* < 0.05); or *d*) the *I*^2^ was large (> 50%, based on the Cochrane’s guide to interpretation of *I*^2^) (Higgins and Green 2011). Review authors were instructed to downgrade only when inconsistent findings reduced confidence in the results in relation to the direction of effect estimates; that is, studies that were inconsistent with respect to the magnitude of an effect (but not in terms of direction of effect estimates) would not be rated down.
Imprecision: Evidence streams were rated down if most studies had small sample sizes and few events, thus leading to wide CIs.
Publication bias: Evidence steams were rated down if we thought that studies were missing from the body of evidence that might result in an overestimate or underestimate of true exposure effects. We used considerations from GRADE guidance for evaluating publication bias, with modifications to reflect the Navigation Guide’s primary concern with underestimating the true effects of existing chemical exposure, in contrast to GRADE’s primary concern of overestimating the true effect of treatments or pharmaceuticals (Guyatt et al. 2011c). These modified considerations for evaluating publication bias included the following: *a*) the body of evidence was dominated by early studies with negative results, particularly studies that were small in size; *b*) studies were uniformly small (particularly when sponsored or funded by industry); *c*) empirical examination of patterns of results (e.g., funnel plots) suggested publication bias; *d*) we were able to obtain results of unpublished studies that demonstrated results different from those of published studies; or *e*) a comprehensive search of the literature was not performed.

Furthermore, the rating of each factor was considered in the context of other limitations. For instance, if the review authors found that two quality issues received borderline decisions (i.e., “risk of bias across studies” and “imprecision”), at least one of the two factors was rated down, as suggested by GRADE (Guyatt et al. 2011d).

The instructions to review authors also contained information on how to evaluate the human body of evidence for upgrading based on consideration of three factors (animal evidence was not eligible for upgrading because of its initial “high” rating); for details, see Supplemental Material, “Instructions for rating the quality and strength of human and nonhuman evidence.” Possible ratings were 0 (no change), +1 (one-level upgrade), or +2 (two-level upgrade):

Large magnitude of effect: GRADE (Guyatt et al. 2011d) recommends rating the evidence stream up by one category (e.g., from “low” to “moderate”) if there were associations with a relative risk (RR) > 2, and up by two categories (for instance, from “low” to “high”) for those with RR > 5. However, there are limitations to using RR to determine magnitude of effect because RR relies on dichotomous exposure scales and outcomes. Although there is no established cutoff for the continuous scales, we evaluated the evidence judiciously to assess whether the magnitude of effect from the human evidence was compelling enough to justify upgrading the evidence.
Dose–response gradient: The evidence stream was rated up if there were consistent dose–response gradients within one or more studies and/or evidence of dose response across the studies in the overall body of evidence.
Confounders minimize the demonstrated effect: The evidence stream was rated up if consideration of plausible residual confounders or biases would only reduce the magnitude of the observed effect, or would suggest a spurious effect when results show no effect. GRADE provides an illustrative example of rating up observational evidence that showed a lack of association between vaccination and autism, which occurred despite empirically confirmed bias that parents of autistic children may be more likely to remember their vaccine experience (Guyatt et al. 2011d). The negative findings despite this form of recall bias suggest rating up the quality of evidence (Guyatt et al. 2011d).

Consistent with GRADE’s approach to evaluating risk of bias across studies (Guyatt et al. 2011e), the review authors were instructed to be conservative in making judgments to downgrade the evidence for all factors (i.e., high confidence in concerns with the body of evidence before rating down). After independently evaluating the quality of the evidence, all authors collectively discussed their evaluations. This discussion between coauthors was extensive and iterative and was carried out over several meetings until a consensus was reached. Specifically, these collective decisions did not involve a “majority vote” or other tallying of perspectives. It was prespecified that discrepancies between the review authors’ judgments that could not be resolved through consensus would be resolved by the senior author (T.J.W.). However, for this case study, review authors were able to agree on a collective consensus for each rating and the arbiter was not necessary. The collective rationale for each decision on each of the factors was documented and recorded.

Rating the strength of the evidence across studies. In systematic reviews in the clinical sciences, only human evidence is considered in a decision, and so there exists no corollary step for integrating multiple streams of evidence in Cochrane or other methods of systematic review in the clinical sciences. We followed guidance from IARC (2006) and used toxicity definitions from the U.S. EPA (1991, 1996) to develop our approach to rate the strength of evidence for the human and nonhuman bodies of evidence (Rooney et al. 2014).

We rated the overall strength of the human and nonhuman evidence separately based on a combination of four considerations, which were developed from existing criteria for evaluating evidence streams (IARC 2006): *a*) quality of body of evidence (i.e., our rating from the previous step), *b*) direction of effect estimates, *c*) confidence in effect estimates (likelihood that a new study would change our conclusion), and *d*) other compelling attributes of the data that may influence certainty (Figure 1). We compared the results of rating the strength of the human and nonhuman evidence to the definitions specified in the Navigation Guide for “sufficient evidence of toxicity,” “limited evidence of toxicity,” “inadequate evidence of toxicity,” or “evidence of lack of toxicity” to select one of these final ratings for each body of evidence. Detailed definitions for each rating can be found elsewhere (Johnson et al. 2014; Koustas et al. 2014).

The review authors independently evaluated the strength of the evidence according to the four considerations specified above to form their opinion of the final rating of strength of evidence for the human and nonhuman evidence as a way to initiate the group discussion and gather all perspectives for consideration. All authors collectively discussed their evaluations in a meeting until a consensus was reached. Specifically, this final rating did not involve a “majority vote” or other tallying of perspectives. It was prespecified that discrepancies between the review authors’ judgments that could not be resolved through consensus would be resolved by the senior author (T.J.W.). However, for this case study, review authors were able to agree on a collective consensus for the final rating for strength of evidence and the arbiter was not necessary. The rationale for our collective decision on each of the criteria and overall ratings was documented and recorded.

### Integration of the Strength of Human and Nonhuman Streams of Evidence

The final step of our review was to integrate the strength of the human and nonhuman streams of evidence into a final concluding statement about PFOA toxicity. We compared the strength of the human and nonhuman evidence ratings from step 3 of the Navigation Guide (Woodruff et al. 2011a), which was based on the method used by IARC (2006), and we used their descriptors of strength of evidence, modified to be relevant for noncarcinogenic assessments (Woodruff and Sutton 2014).

By determining the intersection of the ratings assigned to the human evidence in step 3 with those of the nonhuman evidence (Woodruff et al. 2011a), we came to one of the five possible strength of evidence conclusions about toxicity: “known to be toxic,” “probably toxic,” “possibly toxic,” “not classifiable,” or “probably not toxic.” Importantly, consistent with IARC’s strength of evidence conclusions for cancer end points (IARC 2006), “sufficient evidence of toxicity” in humans would result in a “known to be toxic” final conclusion, regardless of the nonhuman evidence rating. However, “limited evidence of toxicity” in humans could result in a “probably toxic” final conclusion if there was “sufficient evidence of toxicity” in animals or a “possibly toxic” final conclusion if there were “limited,” “inadequate,” or “evidence of lack of toxicity” ratings in animals. The terminology for these conclusions was adapted from IARC’s methods for integrating human and nonhuman evidence (IARC 2006), which in turn were linked to strength of evidence descriptions in use by U.S. EPA (1991, 1996).

## Results

**Figure 2:**
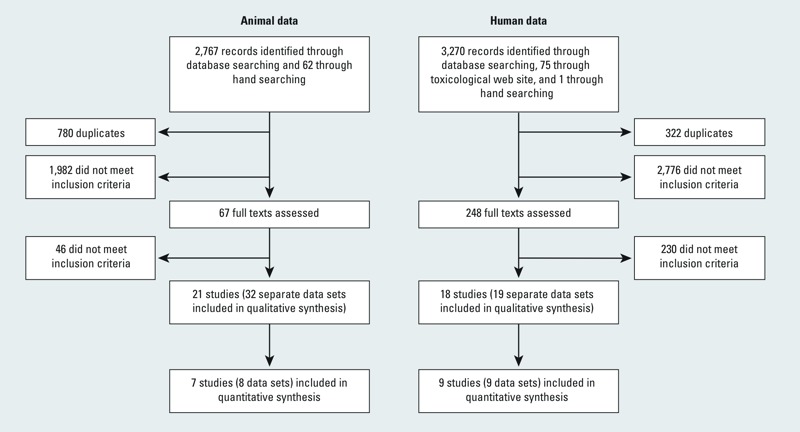
Flowchart showing the literature search and screening process through the systematic review and meta-analysis.

*Included studies*. Our database and hand searches of human literature retrieved a total of 3,024 unique records; of these, we identified a total of 18 relevant studies (which contributed 19 data sets) for analysis (Figure 2). Our database and hand searches of the nonhuman literature retrieved a total of 2,049 unique records; of these, we identified a total of 21 relevant studies (which contributed 32 relevant data sets) for analysis (Figure 2). There were more data sets than studies for both human and nonhuman evidence because some studies contributed multiple data sets, for example, if they measured several relevant outcomes or reported outcomes for different species or populations.

*Risk of bias assessment*. Summaries of the risk of bias determinations for human and nonhuman data are reported by Johnson et al. (2014) and Koustas et al. (2014), respectively. Potential sources of risk of bias that occurred frequently in human studies were confounding, exposure assessment, and conflict of interest. Potential sources of risk of bias that occurred frequently in nonhuman studies were inadequate sequence generation, allocation concealment, and blinding.

*Statistical analysis*. We combined data from nine human studies in a meta-analysis of the effect of PFOA exposure on birth weight. The studies excluded from the meta-analysis were determined to be not combinable with the others because of differences in PFOA exposure scale or outcome statistic (Arbuckle et al. 2012; Halldorsson et al. 2012; Kim SK et al. 2011; Monroy et al. 2008; Nolan et al. 2009; Savitz et al. 2012; Stein et al. 2009). From the meta-analysis, we found an overall estimate of –18.9 g birth weight (BW)/ng/mL increase in serum PFOA (95% CI: –29.8, –7.9) (Johnson et al. 2014). The *I*^2^ was 38%, indicating little heterogeneity between studies that could not be explained by chance; this was further supported by the *Q* statistic (*p* = 0.12). Additional meta-analyses demonstrated that PFOA exposure was also slightly associated with decreases in other fetal growth measures at birth, such as length (*n* = 5; overall estimate, –0.1; 95% CI: –0.1, –0.02), ponderal index (*n* = 4; overall estimate, –0.01; 95% CI: –0.03, 0.01), and head circumference (*n* = 4; overall estimate, –0.03; 95% CI: –0.1, 0.01) (Johnson et al. 2014).

Fifteen of the 21 nonhuman studies were conducted on mammalian species (11 in mice and 4 in rats) and 6 were conducted on nonmammalian species (3 in chickens, 1 in fruit flies, 1 in zebrafish, and 1 in salmon) (Koustas et al. 2014). From an assessment of predetermined considerations regarding study characteristics (e.g., species, route of exposure, method of outcome measurement, time point of outcome measurement), we determined that 7 of these studies (8 data sets), all of which exposed pregnant mice to PFOA via gavage treatment and measured weight of offspring at or soon after birth, were suitable for meta-analysis.

We used the mean pup body weight at birth (± SE) from each of the data sets for all doses < 5 mg/kg BW/day. The dose was limited to focus on effects at lower tested doses and minimize adverse impacts from responses at higher doses, such as litter loss, on the overall estimate. We initially attempted to transform doses tested in animals to human-equivalent serum concentrations for more direct comparisons to the human data; however, a review of the available scientific data produced minimal data that would support such extrapolation. We felt that our limitation to doses < 5 mg/kg BW/day was adequate to ensure relevance of the animal dose–response estimates to humans. Furthermore, by using the slope of the dose–response model for animals, our interpretation assumes that similar increases in exposure would result in the same relative changes in birth weights compared with humans, which we considered reasonable. From the meta-analysis, we found an overall estimate of –0.023 g BW per 1-mg/kg BW/day increase in PFOA dose to pregnant dams (95% CI: –0.029, –0.016) (Koustas et al. 2014). The *I*^2^ test statistic was 0%, indicating no observed statistical heterogeneity between studies that could not be explained by chance; this conclusion was further supported by the *Q* statistic (*p* = 0.73).

We also visually inspected scatter plots of dose–response data for all mammalian and nonmammalian animal data, including data excluded from the meta-analysis (those with study characteristics determined to be too variable to combine) to investigate effects (Koustas et al. 2014). The dose–response data from the eight mammalian data sets included in the meta-analysis showed similar results in the same direction (decreased weight) with mostly statistically significant results. In contrast, the dose–response data for the nine mammalian studies not included in the meta-analysis showed mixed results, generally with lower doses showing increased weight compared with the control group (mostly nonsignificant) and higher doses showing decreased weight (some statistically significant and others not). A qualitative evaluation of data for the nonmammalian studies showed mostly nonstatistically significant increases in body weight (seen in multiple chicken studies, but not in fruit fly or salmon studies, although there was only one study in each species with a small number of tested doses). The length data for nonmammalian studies showed mixed results, including statistically significant decreases in length in fruit flies and zebrafish, but the other two studies (in chickens and salmon) showed insignificant increases in length; these discrepancies, in part, justify our decision to rate the body of nonmammalian studies overall to be of “low” quality (Koustas et al. 2014).

Sensitivity analysis of the human studies demonstrated little change in the overall effect estimate when removing one included study at a time or adding in one excluded study, although the heterogeneity statistics did increase. Sensitivity analysis of the nonhuman studies demonstrated little change in the overall effect estimate or heterogeneity statistic when removing one included study at a time. We originally planned to produce funnel plots of the estimated effects to visually assess the possibility of publication bias, but we did not due to the small number of included studies.

*Quality of the body of evidence*. We evaluated each of the five quality downgrade factors separately for human, nonhuman mammalian, and nonmammalian streams of evidence. We concluded that there was no indication of substantial risk of bias across studies for the available human evidence, particularly when evaluating the studies included in the meta-analysis; thus, we did not downgrade the human evidence for this factor. The majority of nonhuman mammalian studies had “probably high” risk of bias for the allocation concealment and blinding domains. The nonmammalian studies had “probably high” risk of bias for the sequence generation, allocation concealment, and blinding domains. Because these components have been shown empirically to influence study outcomes in experimental animal studies (Bebarta et al. 2003; Landis et al. 2012; Macleod et al. 2004), our group consensus was to downgrade each nonhuman body of evidence by one quality level (–1) for risk of bias across studies.

We concluded there was no indication of substantial indirectness in either the body of available human or nonhuman mammalian evidence. The human studies assessed the population, exposure, and outcomes of interest, as did the nonhuman mammalian evidence, based on empirical evidence that mammalian data can be used as direct evidence for human health inference (Kimmel et al. 1984; U.S. EPA 1996). However, we could not identify a rationale or empirical basis for assuming directness of the nonmammalian body of evidence reviewed in this case study, and in particular, we were concerned about indirectness of the route of exposure (e.g., injection or immersion of eggs in PFOA-containing solution) and developmental differences (*in utero* development vs. egg development) between humans and the nonmammalian model systems. Therefore, we downgraded the nonmammalian evidence one quality level (–1) for indirectness.

We concluded there was no indication of inconsistency in any of the three bodies of evidence. With the exception of two small studies (Fromme et al. 2010; Kim S et al. 2011), results across the human studies were generally consistent in the magnitude and direction of effect estimates. This was further supported by the consistency of the overall meta-analysis results—which were minimally affected by results of any individual study—as determined by sensitivity analysis. For nonhuman mammalian studies, point estimates were generally consistent with overlapping confidence bounds, and meta-analysis results were consistent in the direction of effect estimates and minimally affected by the results of any individual study, as determined by sensitivity analysis. Nonmammalian studies differed based on outcome of measurement (weight vs. length), but results were consistent between comparable studies (similar outcome, species, and exposure route). Therefore, we did not downgrade the quality level for any of the bodies of evidence for inconsistency.

We concluded there was no indication of imprecision in any of the three bodies of evidence. We judged the CIs for both the human and nonhuman mammalian meta-analysis to be sufficiently narrow so as not to warrant downgrading the evidence. Similarly, CIs for the nonmammalian evidence were either sufficiently narrow or, if none were given, the data showed statistically significant responses at high doses, indicating small confidence bounds. The group consensus after evaluating this factor was to not downgrade the quality level for any of the bodies of evidence for imprecision.

We concluded there was no indication of publication bias in any of the three bodies of evidence. The literature search was comprehensive and included strategies to search the grey literature, such as conference abstracts, reports, or other non–peer-reviewed literature. Although we could not ensure that we had identified all unpublished studies, the studies we found had varying sample sizes and funding sources, and none of the unpublished studies we found presented results out of the range of estimates reported by published studies. Without a sufficient number of studies to produce an informative funnel plot to derive evidence about potential missing data, the group consensus was that we did not have substantial evidence to warrant downgrading the quality level for any of the bodies of evidence for publication bias.

We evaluated each of the three upgrade quality factors for human evidence only. We found no compelling evidence to warrant upgrading the evidence based on our prespecified definitions for the three considered factors. We evaluated the human effect estimates judiciously and agreed that the magnitudes of the effect estimates were not compelling enough to justify upgrading the evidence. Although several studies showed some evidence of a dose–response relationship, we agreed that the evidence was not compelling enough across the body of evidence as a whole. We also agreed that there was no evidence to suggest that consideration of plausible residual confounders or biases would reduce the estimated effect. The group consensus after evaluating these factors was to not upgrade the quality level for the human evidence.

A summary of our final decisions for each upgrade/downgrade factor for each of the three bodies of evidence is shown in Table 2. An assessment of these decisions resulted in an overall quality of the human evidence rating of “moderate.” The overall quality rating of the nonhuman mammalian evidence was downgraded from “high” to “moderate” based on the risk of bias across studies. The overall quality rating of the nonmammalian evidence was downgraded from “high” to “low” based on the concerns regarding both the risk of bias across studies and indirectness.

**Table 2:** Summary of the quality ratings given to each body of evidence.

Rating factor	Human	Non­human mammalian	Non­mammalian
Initial rating	Moderate	High	High
Downgrade factors			
Risk of bias across studies	0	–1	–1
Indirectness	0	0	–1
Inconsistency	0	0	0
Imprecision	0	0	0
Publication bias	0	0	0
Upgrade factors			
Large magnitude of effect	0	NA	NA
Dose response	0	NA	NA
Confounding minimizes effect	0	NA	NA
Overall grade	0	–1	–2
Resulting rating	Moderate	Moderate	Low
NA, not applicable. Ratings: 0, no change; –1, decrease rating by one level; –2, decrease rating by two levels.			

*Strength of the body of evidence rating*. We rated the overall strength of the human and nonhuman bodies of evidence separately based on the four considerations: *a*) quality of the body of evidence, *b*) direction of effect estimates, *c*) confidence in effect estimates (likelihood that a new study would change our conclusion), and *d*) other compelling attributes of the data that may influence certainty. Because the quality of the nonmammalian evidence was rated “low,” whereas the nonhuman mammalian data were rated “moderate,” we made the decision to carry forth only the higher quality nonhuman mammalian body of evidence for evaluating strength of evidence. This is consistent with GRADE recommendations: When high-quality data are available for decision making, one does not need to incorporate low quality data (Balshem et al. 2011).

We rated the quality of body of evidence for both human and nonhuman evidence as “moderate,” as discussed above. The direction of effect estimates for both human and nonhuman evidence was assessed by evaluating available data across individual studies as well as using results from the meta-analyses. We concluded that there was similar evidence of an association between decreased birth weight and increased exposures to PFOA for both evidence streams.

We evaluated the confidence in effect estimates using slightly different approaches for each body of evidence. For the human evidence, we used an ad hoc approach of quantitatively evaluating the potential impact of including a new hypothetical study in the overall meta-analysis result. We considered several scenarios of adding a hypothetical study with characteristics similar to those in our included human studies to determine what effect estimates would be needed to alter the interpretation of our final meta-analysis result. Comparing this with the effect estimates of our included human studies, we decided that it seemed unlikely that another human study would find such associations. More details, including the quantitative estimates, are reported by Johnson et al. (2014). For the nonhuman evidence, we determined that our confidence in the effect estimates was high because the results among nonhuman mammalian experimental studies were similar and demonstrated overlapping CIs across different studies (Koustas et al. 2014). Finally, we did not identify any other compelling attributes of the data that would influence our certainty in the estimates. In particular, we considered a hypothesis proposed in the literature whereby women who had smaller babies had higher measures of PFOA due to a lower glomerular filtration rate as a result of lower plasma volume expansion (Verner et al. 2014). We evaluated the supporting scientific evidence for this hypothesis in the context of our final conclusion of this review and decided that it did not undermine our findings for several reasons.

A summary of our strength of evidence determinations for each consideration for human and nonhuman evidence is reported by Johnson et al. (2014) and Koustas et al. (2014), respectively. We compared these determinations with the definitions to evaluate the overall strength of each body of evidence (Johnson et al. 2014; Koustas et al. 2014). Our consensus for the human evidence was that the overall quality of evidence was “moderate,” and we had a high level of confidence in an association between decreased birth weight and increased exposures to PFOA. Comparing our consensus on these considerations to the definitions of “sufficient evidence of toxicity,” “limited evidence of toxicity,” “inadequate evidence of toxicity,” or “evidence of lack of toxicity,” we agreed that *a*) our findings met the definitions for “sufficient evidence of toxicity” (i.e., a positive relationship was observed between exposure and outcome where chance, bias, and confounding could be ruled out with reasonable confidence); *b*) the available evidence included results from one or more well-designed, well-conducted studies; and *c*) the conclusion was unlikely to be strongly affected by the results of future studies.

Our consensus for the nonhuman studies was that the overall body of evidence was “moderate,” and we had a high level of confidence in an association between decreased birth weight and increased exposures to PFOA. We agreed that *a*) our findings for the nonhuman (mammalian) studies met the definitions for “sufficient evidence of toxicity” (i.e., a positive relationship was observed between exposure and adverse outcome in multiple studies or a single appropriate study in a single species); *b*) the available evidence included results from one or more well-designed, well-conducted studies; and *c*) the conclusion was unlikely to be strongly affected by the results of future studies.

Our final conclusion for the overall strength of evidence was that there was “sufficient evidence of toxicity” in humans and “sufficient evidence of toxicity” in nonhuman mammals to support a judgment that exposure to PFOA affects fetal growth.

*Integrating the evidence across evidence streams*. We integrated our evidence rating of “sufficient evidence of toxicity” for the human and the nonhuman evidence and concluded that PFOA should be classified as “known to be toxic.”

## Discussion

The application of the Navigation Guide systematic review methodology demonstrated a novel method for integrating diverse sources of toxicity data to reach strength of evidence conclusions for noncancer health effects in environmental health. Application of the method produced a clear and concise conclusion by the authors of this review: that exposure to PFOA is “known to be toxic” to human reproduction and development based on sufficient evidence of decreased fetal growth in both human and nonhuman mammalian species.

For the human data, we concluded that there was sufficient evidence of an association based on *a*) a transparent collective rating of the evidence as “moderate” quality; *b*) a meta-analysis estimating a reduction in birth weight in relation to PFOA exposure for which confidence bounds were sufficiently narrow and did not include zero; and *c*) our confidence that it would be unlikely for a new study to have an effect estimate that could substantially change the overall effect estimate of the meta-analysis (Johnson et al. 2014). Similarly, we concluded for the nonhuman data that there was sufficient evidence of an association based on *a*) a transparent collective rating of the available nonhuman mammalian evidence as “moderate” quality; *b*) a meta-analysis showing a reduction in birth weight in relation to PFOA dose for which confidence bounds were narrow and did not include zero; and *c*) our confidence that the conclusion was unlikely to be strongly affected by the results of future studies (Koustas et al. 2014).

In applying the Navigation Guide methodology to this case study, we found that the definitions used to rate the quality and strength of the evidence drive the final strength of evidence statement. Although the domains and factors used for rating quality of evidence were derived from methods applied in the clinical sciences (Guyatt et al. 2008; Higgins and Green 2011), there is no precedent for defining and integrating strength of evidence conclusions among different evidence streams in the clinical sciences. Our definitions for strength of evidence and the process for integration of the evidence streams were derived from current practices in use by IARC (2006) and the U.S. EPA (1991, 1996). Notably, although the Navigation Guide currently requires “sufficient” human evidence for a chemical to be rated as “known to be toxic,” this requirement may be revised in future case studies to align with other established methods in environmental health in which this requirement is not necessary (IARC 2006; Rooney et al. 2014; U.S. EPA 1991, 1996). Given that the risk of bias criteria, the factors used to rate quality across a body of evidence, and the considerations for rating strength of evidence underlie the final integration step, research to deepen our knowledge of the relative and absolute impact of each of these criteria, factors, and considerations in the final strength of evidence rating is currently a critical need.

We found that prespecified definitions made rating the evidence at hand efficient and transparent. First, establishing precise definitions ensured that we were all using the same rules to apply our judgment and ensured that our collective decisions were transparent and explicit even to ourselves. Second, determining prespecified definitions encouraged us to actively take into account the sources of data and the evidence necessary to support different conclusions regarding weight and strength of evidence, and to identify how to establish scientifically valid definitions. The definitions we used in this first case study can guide development of definitions for future case studies; however, they are not rigid and they can potentially be refined to apply to any particular question and available body of evidence at hand.

Although the protocol predefined many of the guidelines for making decisions, we found we could not anticipate all decision points beforehand. For example, we did not anticipate that our search would retrieve data on such diverse nonmammalian model systems (such as zebrafish and chickens), and during the analysis, we had to interpret the heterogeneity and relevance of these data to human health. In another example, in following recommendations from GRADE, we defined the factor for upgrading the quality of evidence based on large magnitude of effect as associations with an RR > 2 (+1 upgrade to the evidence) or an RR > 5 (+2 upgrade to the evidence). However, the data from the human evidence were more amenable to a meta-analysis performed on a continuous scale; therefore, we did not have RRs to compare using this definition. Furthermore, RRs on a scale of 2 or 5 for nonoccupational studies are a rarity in the field of environmental health because of the relatively low levels of exposure to environmental contaminants (Taubes 1995). Although this is generally an accepted cutoff for GRADE, the definition of a large magnitude of effect will require adjustment based on the nature and extent of the available evidence. Additional consideration may also be required because the size of RR estimates is dependent on the study authors’ selection of the comparator group. Therefore, the definition of a large magnitude of effect may need adjustment based on the design of included studies and the specific biological outcomes.

For this case study, we decided beforehand to define the “inconsistency” factor to rate down each body of evidence if studies showed widely different estimates of effect, but we did not include a consistency factor to rate up each body of evidence for the converse scenario. Our goal was to ensure that all bodies of evidence would be evaluated for consistency; therefore, because the nonhuman evidence was not assessed for upgrade factors because it started at “high,” we included inconsistency as a downgrade. This is consistent with GRADE recommendations for evaluating inconsistency for human evidence (Guyatt et al. 2011b). This definition of the inconsistency factor is not rigid and can be adjusted for future case studies. One example is the recent proposal by the National Toxicology Program’s Office of Health Assessment and Translation for which systematic review and evidence integration for health assessments instead includes “consistency” as a factor that increases confidence in the body of evidence (National Toxicology Program 2013) compared with our use of inconsistency as a downgrade factor. The approach to categorizing these factors may change, but the underlying consistency and transparency of each approach to evaluate the bodies of evidence is what is most important.

In recent years, several scientists have hypothesized that maternal and fetal physiology may influence measured blood levels indicating an exposure; in particular for PFOA and reduced birth weight, these associations may be due to reverse causality whereby women who have smaller babies have higher measures of PFOA as a result of a lower glomerular filtration rate caused by lower plasma volume expansion (Loccisano et al. 2013; Savitz 2007; Whitworth et al. 2012). If this reverse causality hypothesis were true, it would explain some or all of the relationship observed in human cross-sectional studies documenting an inverse association between fetal growth and prenatal exposure to exogenous chemicals with renal clearance, such as PFOA.

We considered this hypothesis and its supporting scientific evidence in the context of the final conclusion of our review and decided that it did not undermine our findings for two reasons. First, this hypothesis is not relevant to associations found in animal studies. In our review of PFOA, the experimental animal evidence was robust and mirrored the human evidence, lending support for the association between PFOA exposure and low birth weight (Koustas et al. 2014). Second, we systematically reviewed the literature for evidence of the relationship between birth weight and maternal glomerular filtration rate (see Supplemental Material, “List of studies included in systematic review of the relationship between birth weight and maternal glomerular filtration rate”) and concluded that there is currently insufficient evidence to support the reverse causality hypothesis for associations between fetal growth and maternal glomerular filtration rate in humans. Additional research is needed to confirm or disprove this hypothesis. Thus, although we cannot disprove reverse causality, we have found no conclusive evidence currently available to justify altering our conclusions regarding the strength of human evidence. However, review authors were cognizant of the potential for these physiological factors associated with pregnancy to account for the negative association of PFOA with low birth weight. A preliminary study based on physiologically based pharmacokinetic (PBPK) modeling of a meta-analysis of seven published epidemiology studies suggested that a portion of the association between PFOA and low birth weight was attributed to confounding by glomerular filtration rate (Verner et al. 2014). Another study investigating hematologic changes and pregnancy outcomes similarly showed that low hemoglobin in late pregnancy was associated with low birth weight, but the association disappeared after adjusting for plasma volume (Whittaker et al. 1996). However, there remains a lack of human evidence that this is indeed the case for external chemical exposures. Although the reverse causation hypothesis is reasonable and warrants further investigation, without stronger evidence—and in light of the strength of the animal data—we believe that downgrading the final conclusion for “sufficient” for the human evidence was not justifiable at this time.

Ultimately, our application of the Navigation Guide approach led to a clear and concise concluding statement, resulting from a systematic and transparent review of the literature developed from comprehensive and transparent methods used in the clinical sciences that have been demonstrated to reduce bias (Antman et al. 1992; Higgins and Green 2011). This is unique to the Navigation Guide systematic review methodology as well as the method under development by the National Toxicology Program (National Toxicology Program 2013; Rooney et al. 2014). A comparison of our results with those of previous reviews of PFOA (DeWitt et al. 2009; Hekster et al. 2003; Jensen and Leffers 2008; Kennedy et al. 2004; Kudo and Kawashima 2003; Lau et al. 2004, 2007; Lindstrom et al. 2011; Olsen et al. 2009; Post et al. 2012; Steenland et al. 2010; White et al. 2011) showed that the application of the Navigation Guide provided more transparency about the steps taken in the review and a consistent path to a clear answer compared with methods of expert-based narrative review that are currently employed in environmental health (Woodruff and Sutton 2014).

Adami et al. proposed a framework to combine the toxicological and epidemiological evidence to establish causal inference (Adami et al. 2011; Simpkins et al. 2011). Although similar to the Navigation Guide in seeking greater transparency overall in research synthesis and striving to integrate human and nonhuman evidence into a final conclusion, the methods differ in substantive, fundamental ways. Specifically, the Adami method does not conform to key features of systematic review methodologies, that is, a prespecified protocol, a comprehensive search strategy, a risk of bias assessment, and data analysis. Moreover, the Navigation Guide, modeled in accordance with the IARC framework (IARC 2006), gives primacy to the strength of the human evidence stream in the absence of an established mode of action; however, in the Adami method, conclusions about a body of evidence rests explicitly on whether or not a mode of action relevant to humans has been established by the toxicological evidence: that is, if the mode of action established in animal models is considered to be irrelevant to humans, then the biological plausibility of the effect observed in humans through the proposed mode of action is considered to be “highly unlikely.” More research targeted on identifying and evaluating the utility, transparency, and robustness of different methods—including the questions they are suitable for answering—will be useful in the future as the application of improved methods becomes more widespread (Krauth et al. 2013).

## Limitations

One benefit of our adoption of the IARC approach is that it was transparent and simple to integrate the evidence from human and nonhuman bodies of available evidence once we rated each stream’s strength of evidence separately. However, this meant that quantitative evaluations of the effect estimates for each body of evidence were kept separate and not integrated earlier on in the process. There has been much discussion recently in several research fields to utilize quantitative methods that can integrate diverse sources of data, such as human and nonhuman toxicity evidence, into a single quantitative model that can account for the different sources of data and expected contribution of each data set to the evidence for human toxicity (DuMouchel and Harris 1983; Jones et al. 2009; Peters et al. 2005). Future investigation into methods for quantitatively integrating these diverse sources of data (e.g., in a hierarchical Bayesian model) is warranted and would be an important contribution to advancing strength of evidence conclusions in environmental health.

The nomenclature of the overall strength of the human evidence (i.e., the terms “known,” “probably,” and “toxic”) generally had differing connotations among review authors despite agreement on the underlying definitions that supported the final conclusion. Some of the review authors found “known to be toxic” to be an accurate descriptor of the body of evidence, whereas others felt the descriptor “probably toxic” was more appropriate. Our discussions of the variability of our own subjective reactions to “known” and “probably” emphasized the need for further delineation of prespecified objective criteria for the strength of the evidence definitions.

Our different subjective reactions over terminology were resolved by focusing our discussion on the definitions we had established for each strength of evidence rating (Johnson et al. 2014; Koustas et al. 2014). From this discussion, ultimately all authors agreed with the final concluding statement. However, such consensus may not always be possible because the available evidence is not always clear-cut. Conclusions about the strength of the evidence regarding toxicity must be made for regulatory purposes, for choosing less toxic alternatives, and/or for other purposes; as in the clinical sciences, complete agreement on the strength of the evidence should not be a criterion for enabling government agencies, professional societies, health care organizations, or others to make a determination. An example of this is California’s Proposition 65, a voter-approved initiative that gives the state authority to classify chemicals deemed to cause cancer, birth defects, or reproductive health effects (California Office of Environmental Health Hazard Assessment 2013). One mechanism by which chemicals are added to the list is that either of two independent scientific committees concludes that the chemical has clearly been shown to cause these adverse health effects. Consensus is not required from both committees, and even within an individual committee the vote to add a chemical to the list does not have to be unanimous: For example, the recent addition of tris(1,3-dichloro-2-propyl) phosphate (TDCPP) was determined based on a 5–1 vote in one committee (California Office of Environmental Health Hazard Assessment 2011).

Addressing a lack of consensus in the interpretation of scientific evidence reinforced a key methodological strength of systematic reviews—transparent definitions and documentation of the basis of a conclusion—so the rationale for the final toxicity statement can be readily interpreted and/or contested by outside entities. In particular, it is critical to provide both a final recommendation and the documentation and justification leading to this conclusion. In addition, we anticipate that readers will have their own subjective connotations and reactions to our concluding statement. Although our nomenclature (i.e., “known,” “possibly,” and so on) was developed by modifying the nomenclature used by IARC (2006) and the U.S. EPA (1991, 1996) to classify carcinogens for many years, the use in this context—adapted to be more broadly applicable to both carcinogens and noncarcinogens—and its utility to decision makers are untested.

Specifically, there is currently no consensus in environmental health on how to name and communicate the strength of the evidence, and indeed there are many examples of similar terms that are commonly used to characterize varying strengths of evidence; for example, terms used to describe “moderate” evidence include “balance of evidence,” “balance of probabilities,” “reasonable grounds of concern,” and “strong possibility” (Gee 2008). Research related to climate change has shown that the public consistently misinterprets probabilistic statements such as “unlikely” or “very unlikely,” used in Intergovernmental Panel on Climate Change reports, and there are large individual differences in the interpretation of the statements that are associated with the public’s views and beliefs on climate change (Budescu et al. 2012). Research on better ways to communicate uncertainty is critical, and discussion of improved communication needs to include the users of the information, such as policy makers and the public.

Our case study was limited to human and nonhuman animal data. There is a need to expand the scope of the Navigation Guide systematic review method to incorporate the results of *in vitro* studies and other modern methods of toxicology testing into the reviewed evidence stream. It is critical to develop such approaches because *in vitro* and other model systems and types of data will play an increasingly important role in the regulatory sphere as advances in technology allow for the rapid production of large quantities of data, such as those used in high-throughput screening (NRC 2007; U.S. Food and Drug Administration 2011).

Furthermore, our first case study in which we applied the Navigation Guide ended with step 3, and we did not make a final recommendation about what to do about the science. Step 4 of the Navigation Guide is where the conclusion regarding toxicity is combined with additional information such as exposure prevalence, consideration of available alternatives, values, and preferences to determine the final recommendation for public health protection. The Navigation Guide method allows for substances “known to be toxic” to have discretionary recommendations, and substances “possibly toxic” to have strong recommendations, depending on these and other potential factors. Although we did not address step 4 in this case study because of resource limitations, carrying a case study through all of the Navigation Guide steps is a research need for the future, which will demonstrate how to apply the Navigation Guide in risk management decisions.

Finally, exposures to environmental contaminants that lead to chronic disease or adverse reproductive and developmental health outcomes are complex and poorly understood. Such harm can be irreversible and can span across generations, making a strong case for timely decision making and actions to prevent harm. However, having limited data or multiple studies of varying quality and findings can often hinder the ability to take such action. Criteria for evaluating diverse sources of scientific evidence to support action on the science is lacking and is therefore a critical unmet research need (Krauth et al. 2013).

## Conclusion

Our case study demonstrates an application of the Navigation Guide to apply the rigor and transparency of systematic review methodology from the clinical sciences to make strength of evidence conclusions in environmental health. Here, we combined the strength of evidence ratings from the nonhuman (Koustas et al. 2014) and human (Johnson et al. 2014) evidence following the framework proposed in the Navigation Guide (Woodruff et al. 2011a), and review authors came to the final conclusion that exposure to PFOA is “known to be toxic” to human reproduction and development based on sufficient evidence of decreased fetal growth in both human and nonhuman mammalian species. This demonstrated the utility of the Navigation Guide to systematically evaluate the available evidence to answer questions relevant to environmental health. We anticipate that future applications of the Navigation Guide methodology to additional case studies will refine and improve the approach, contributing to the ultimate goal of supporting timely evidence-based decisions and recommendations for the prevention of harm to public health.

## Supplemental Material

(204 KB) PDF
