# Supplementary material for: The Navigation Guide—Evidence-Based Medicine Meets Environmental Health: Integration of Animal and Human Evidence for PFOA Effects on Fetal Growth
Source: Environ Health Perspect. 2014 Jun 25;122(10):1040–51. doi: 10.1289/ehp.1307923 (PMC4181930; doi:10.1289/ehp.1307923)
Supplement: (204 KB) PDF [file ehp.1307923.s001.508.pdf]

Supplemental Material

The Navigation Guide—Evidence-Based Medicine Meets Environmental Health: Integration of Animal and Human Evidence for PFOA Effects on Fetal Growth

Juleen Lam, Erica Koustas, Patrice Sutton, Paula I. Johnson, Dylan S. Atchley, Saunak Sen, Karen A. Robinson, Daniel A. Axelrad, and Tracey J. Woodruff

Table of Contents **Page**

Navigation Guide Workgroup Members 2

Instructions for rating the quality and strength of human and non-human evidence 3

I. Rate the Quality of Evidence 4

II. Rate the Strength of Evidence 12

III. Combine Strength of Evidence For Human and Non-human Evidence 12

List of studies included in systematic review of the relationship between birth weight and maternal glomerular filtration rate 13

References 16

Navigation Guide Workgroup Members

Tracey J. Woodruff (University of California, San Francisco)

Patrice Sutton (University of California, San Francisco)

Vincent James Cogliano (International Agency for Research on Cancer)

Kate Guyton (Environmental Protection Agency [EPA])

Julia Quint (Retired, California Department of Public Health)

Lauren Zeise (CA-EPA)

Judith Balk (University of Pittsburgh [UP])

Lisa Bero (UCSF)

Jeanne Conry (Kaiser Permanente, American College of Obstetricians and Gynecologists District IX)

Daniel M. Fox (Emeritus, Milbank Memorial Fund)

David Gee (European Environmental Agency)

Rivka Gordon (Association of Reproductive Health Professionals [ARHP])

Sarah Janssen (Natural Resources Defense Council)

Beth Jordan (ARHP)

Victoria Maizes (University of Arizona)

Mark Miller (UCSF)

Michele Ondeck (UP)

Karen Pierce (San Francisco Department of Public Health)

Pablo Rodriguez (Brown Medical School and Women & Infants Hospital of Rhode Island)

Heather Sarantis (Collaborative on Health and the Environment)

Ted Schettler (Science and Environmental Health Network)

Sandy Worthington (Planned Parenthood Federation of America)

Instructions for rating the quality and strength of human and non-human evidence

1. Co-authors will independently review the final data and independently rate the quality of evidence according to *a priori* criteria set forth in the protocol.

2. Co-authors will compare their results. Any discrepancies between the co-authors' decisions will be resolved through discussion. The senior author (TW) will be the ultimate arbiter of the discrepancies that cannot be resolved through consensus among the co-authors. The final judgments of all reviewers will be documented.

3. The initial quality level of non-human experimental data is considered "high" consistent with GRADE guidelines for rating experimental studies (i.e., randomized controlled trials). The initial quality level of human observational data is considered "moderate". This is in contrast to GRADE guidelines, developed for clinical interventions, which assign observational studies an initial rating of "low" quality (Balshem et al. 2011). There is variability in the quality of studies, however, and not all observational studies may be low quality (Viswanathan et al. 2012). In environmental health, human observational data are the "best" data available for decision-making, and in this regard they are comparable to human randomized controlled trials (RCTs) in the clinical sciences. Because ethics virtually precludes human RCTs in environmental health, beginning human observational studies at "moderate" quality captures the value of these data relative to what data are available. In addition, human observational studies are recognized as being a reliable source of evidence in the clinical sphere, as not all healthcare decisions are, or can be, based on RCTs; (Institute of Medicine et al. 2008) recognition of the absolute value of human observational data evidence-based to clinical decision-making is also increasing (Peterson 2008; Halvorson 2008).

4. "Fetal growth" is the outcome being assessed in this review.

    - In humans, the outcome fetal growth includes all the following measures: birth weight, birth length, head circumference, and ponderal index; all of these measures are sufficiently similar to rate together as a measure of the same outcome.

    - In non-human mammalians, the outcome "fetal" growth includes all the following measures: "Fetal" data, which refers to when outcome measurements are taken from progeny near-term (i.e., E18 for mice, E21 for rats). "Pup" data, which refers to when outcome measurements are taken from progeny at or soon after birth.

    - In non-human non-mammalians, the outcome fetal growth is equivalent to "embryonic" growth and includes measures of weight, length or volume, depending on the model system.

5. For the purpose of the PFOA case study, there are 3 populations for which we are rating the quality of evidence for PFOA's effect on fetal growth: (1) the quality of human evidence for fetal growth; (2) the quality of mammalian animal evidence for fetal growth; and (3) the quality of non-human, non-mammalian evidence for fetal growth.

6. There are 5 categories that can lead to **downgrading** quality of evidence for an outcome: risk of bias (study limitations); indirectness; inconsistency; imprecision; and publication bias. According to GRADE, these 5 categories address nearly all issues that bear on the quality of evidence (Balshem et al. 2011). GRADE states that these categories were arrived at through a case- based process by members of GRADE, who identified a broad range of issues and factors related to the assessment of the quality of studies. All potential factors were considered, and through an iterative process of discussion and review, concerns were scrutinized and solutions narrowed by consensus to these five categories. GRADE also defines 3 categories that can lead to upgrading quality of evidence for an outcome: large effect; confounding would minimize effect; and dose response.

7. While GRADE specifies systematic review authors consider quality of evidence under a number of discrete categories and to either rate down or not on the basis of each category, they also state that rigid adherence to this approach ignores the fact that quality is actually a continuum and that an accumulation of limitations across categories can ultimately provide the impetus for rating down in quality (Guyatt et al. 2011a). Thus authors who decide to rate down quality by a single level will specify the one category most responsible for their decision while documenting all factors that contributed to the final decision to rate down quality.

8. The quality of evidence rating for human and non-human data will be translated into strength of evidence ratings for each stream of evidence.

9. The strength of evidence for human and non-human data will be combined into an overall statement of toxicity, i.e., known to be toxic to fetal growth; probably toxic to fetal growth; possibly toxic to fetal growth; known to be not-toxic to fetal growth.

I. Rate the Quality of Evidence

Each of the categories to consider in downgrading or upgrading the evidence is described in detail, below. Please record your results on the chart at the end of each category, including a brief explanation for your ratings.

Category 1. Rate the Quality of Study Limitations (Risk of Bias) (Guyatt et al. 2011b)

Possible ratings: 0=no change; -1 or -2 downgrade 1 or 2 levels.

The evidence from studies can be rated down if most of the relevant evidence comes from studies that suffer from a high risk of bias. Risk of bias is rated by outcome across studies. Study limitations for each outcome for individual studies and across studies are summarized in the heat maps.

GRADE outlines the following principles for moving from risk of bias in individual studies to rating quality of evidence across studies.

1. In deciding on the overall quality of evidence, one does not average across studies (for instance if some studies have no serious limitations, some serious limitations, and some very serious limitations, one does not automatically rate quality down by one level because of an average rating of serious limitations). Rather, judicious consideration of the contribution of each study, with a general guide to focus on the high-quality studies is warranted.

   (Note: Limitations to GRADE's risk of bias assessments as stated by GRADE: "First, empirical evidence supporting the criteria is limited. Attempts to show systematic difference between studies that meet and do not meet specific criteria have shown inconsistent results. Second, the relative weight one should put on the criteria remains uncertain. The GRADE approach is less comprehensive than many systems, emphasizing simplicity and parsimony over completeness. GRADE's approach does not provide a quantitative rating of risk of bias. Although such a rating has advantages, we share with the Cochrane Collaboration methodologists a reluctance to provide a risk of bias score that, by its nature, must make questionable assumptions about the relative extent of bias associated with individual items and fails to consider the context of the individual items.")

2. This judicious consideration requires evaluating the extent to which each study contributes toward the estimate of magnitude of effect. This contribution will usually reflect study sample size and number of outcome events larger studies with many events will contribute more, much larger studies with many more events will contribute much more.

3. One should be conservative in the judgment of rating down. That is, one should be confident that there is substantial risk of bias across most of the body of available evidence before one rates down for risk of bias.

4. The risk of bias should be considered in the context of other limitations. If, for instance, reviewers find themselves in a close-call situation with respect to two quality issues (risk of bias and, say, precision), GRADE suggests rating down for at least one of the two.

5. Notwithstanding the first four principles, reviewers will face close-call situations. You should acknowledge that you are in such a situation, make it explicit why you think this is the case, and make the reasons for your ultimate judgment apparent.

Type of study	Risk of bias (study limitations) rating	Rationale for your judgment
Human		
Non-human mammalian		
Non-human non-mammalian		

Category 2. Rate Indirectness of Evidence

Possible ratings: 0=no change; -1 or -2 downgrade 1 or 2 levels.

Quality of evidence (your confidence in estimates of effect) may decrease when substantial differences exist between the population, the exposure, or the outcomes measured in research studies under consideration in the review.

Evidence is direct when it directly compares the exposures in which we are interested when applied to the populations in which we are interested and measures outcomes important to the study question (in GRADE the outcomes must be important to patients).

Based on GRADE (Guyatt et al. 2011c) (as modified to reflect our "PECO" instead of "PICO" question), evidence can be indirect in one of three ways. (Note: GRADE includes a fourth type of indirectness that occurs when there are no direct (i.e., head-to-head) comparisons between two or more interventions of interest. This criterion is not relevant to our study question related to toxicity of PFOA; it could be relevant to future case studies.)

1. The population studied differs from the population of interest (the term applicability is often used for this form of indirectness). **Please note the Navigation Guide's *a priori* assumption is that mammalian evidence of a health effect/lack of health effect is deemed to be direct evidence of human health with regards to directness of the population**. This is a marked departure from GRADE (note: According to GRADE, in general, GRADE rates animal evidence down two levels for indirectness. They note that animal studies may, however, provide an important indication of drug toxicity. GRADE states, "Although toxicity data from animals does not reliably predict toxicity in humans, evidence of animal toxicity should engender caution in recommendations." However, GRADE does not preclude rating non-human evidence as high quality. They state, "Another type of nonhuman study may generate high- quality evidence. Consider laboratory evidence of change in resistance patterns of bacteria to antimicrobial agents (e.g., the emergence of methicillin-resistant staphylococcus aureus-MRSA). These laboratory findings may constitute high-quality evidence for the superiority of antibiotics to which MRSA is sensitive vs. methicillin as the initial treatment of suspected staphylococcus sepsis in settings in which MRSA is highly prevalent"), based on empirical evidence in environmental health science that the reliability of experimental animal (mammalian) data for reproductive and developmental health has been well established though multiple studies of concordance between mammalian animals and humans after exposure to a variety of chemical agents (Hemminki and Vineis 1985; Nisbet and Karch 1983; Kimmel et al. 1984; Nemec et al. 2006; Newman et al. 1993). Presently, there is no example of a chemical agent that has adversely affected human reproduction or development

but has not caused the same or similar adverse effects in mammalian animal models (Kimmel et al. 1984). The National Academy of Sciences (NAS) has recognized the importance of animal data in identifying potential developmental risks. According to the NAS, studies of comparison between developmental effects in animals and humans find that "there is concordance of developmental effects between animals and humans and that humans are as sensitive or more sensitive than the most sensitive animal species (National Research Council (U.S.) Committee on Developmental Toxicology and National Research Council (U.S.) Commission on Life Sciences 2000)." GRADE states that in general, one should not rate down for population differences unless one has compelling reason to think that the biology in the population of interest is so different than the population tested that the magnitude of effect will differ substantially. According to GRADE, most often, this will not be the case. In applying this GRADE principle to the Navigation Guide, non-human evidence would be rated down as indirect when it is a biologically inappropriate non-human model system for the health outcome under study.

2. The intervention (exposure) tested may differ from the exposure of interest, i.e., a difference in the chemical, route and/or dose. Decisions regarding indirectness of populations and exposure depend on an understanding of whether biological or social factors are sufficiently different that one might expect substantial differences in the magnitude of effect. GRADE also states, "As with all other aspects of rating quality of evidence, there is a continuum of similarity of the intervention that will require judgment. It is rare, and usually unnecessary, for the intended populations and interventions to be identical to those in the studies, and we should only rate down if the differences are considered sufficient to make a difference in outcome likely."

3. Outcomes may differ from those of primary interest, for instance, surrogate outcomes that are not themselves important, but measured in the presumption that changes in the surrogate reflect changes in an important outcome. The difference between desired and measured outcomes may relate to time frame. When there is a discrepancy between the time frame of measurement and that of interest, whether to rate down by one or two levels will depend on the magnitude of the discrepancy. Another source of indirectness related to measurement of outcomes is the use of substitute or surrogate endpoints in place of the exposed population's important outcome of interest. In general, the use of a surrogate outcome requires rating down the quality of evidence by one, or even two, levels. Consideration of the biology, mechanism, and natural history of the disease can be helpful in making a decision about indirectness. Surrogates that are closer in the putative causal pathway to the adverse outcomes warrant rating down by only one level for indirectness. GRADE states that rarely, surrogates are sufficiently well established that one should choose not to rate down quality of evidence for indirectness. In general, evidence based on surrogate outcomes should usually trigger rating down, whereas the other types of indirectness will require a more considered judgment.

**Type of study**	**Indirectness rating**	**Rationale for your judgment**
Human		
Non-human mammalian		
Non-human non-mammalian		

Category 3. Rate Inconsistency of Evidence

Possible ratings: 0=no change; -1 or -2 downgrade 1 or 2 levels.

According to Cochrane, "when studies yield widely differing estimates of effect (heterogeneity or variability in results) investigators should look for robust explanations for that heterogeneity. … When heterogeneity exists and effects the interpretation of results, but authors fail to identify a plausible explanation, the quality of the evidence decreases."

Based on GRADE (Guyatt et al. 2011d), **a body of evidence is not rated up in quality if studies yield consistent results**, **but may be rated down in quality if inconsistent**. Their stated reason is that a consistent bias will lead to consistent, spurious findings.

GRADE suggests rating down the quality of evidence if large inconsistency (heterogeneity) in study results remains after exploration of a priori hypotheses that might explain heterogeneity. Judgment of the extent of heterogeneity is based on similarity of point estimates, extent of overlap of confidence intervals, and statistical criteria. GRADE's recommendations refer to inconsistencies in effect size, specifically to relative measures (risk ratios and hazard ratios or odds ratios), not absolute measures.

Based on GRADE, reviewers should consider rating down for inconsistency when:

1. Point estimates vary widely across studies;
2. Confidence intervals (CIs) show minimal or no overlap;
3. The statistical test for heterogeneity-which tests the null hypothesis that all studies in a meta-analysis have the same underlying magnitude of effect- shows a low P-value;
4. The I^2 -which quantifies the proportion of the variation in point estimates due to among-study differences-is large. (I.e., the I^2 index quantifies the degree of heterogeneity in a meta-analysis).

GRADE states that inconsistency is important **only when it reduces confidence in results in relation to a particular decision**. Even when inconsistency is large, it may not reduce confidence in results regarding a particular decision. For example, studies that are inconsistent related to the magnitude of a beneficial or harmful effect (but are in the same direction) would not be rated down; in instances when results are inconsistent as to whether there is a benefit or harm of treatment, GRADE would rate down the quality of evidence as a result of variability in results, because the meaning of the inconsistency is so relevant to the decision to treat or not to treat.

Type of study	Inconsistency rating	Rationale for your judgment
Human		
Non-human mammalian		
Non-human non-mammalian		

Category 4. Rate Imprecision of Evidence

Possible ratings: 0=no change; -1 or -2 downgrade 1 or 2 levels.

Cochrane states that when studies have few participants and few events, and thus have wide confidence intervals (CIs), authors can lower their rating of the quality of evidence. These ratings of precision are made as judgments by review authors.

GRADE defines evidence quality differently for systematic reviews and guidelines. For systematic reviews, quality refers to confidence in the estimates of effect. For guidelines, quality refers to the extent to which confidence in the effect estimate is adequate to support a particular decision (Guyatt et al. 2011). For the purpose of step 3 of Navigation Guide, we will use the systematic review definition, because the decision phase does not occur until step 4 when recommendations for prevention are made. Thus, when reviewing the data for imprecision, evaluate your confidence in the estimate of the effect.

According to GRADE, to a large extent, CIs inform the impact of random error on evidence quality. When considering the quality of evidence, the issue is whether the CI around the estimate of exposure effect is sufficiently narrow. If it is not, GRADE rates down the evidence quality by one level (for instance, from high to moderate). If the CI is very wide, GRADE might rate down by two levels.

Type of study	Imprecision rating	Rationale for your judgment
Human		
Non-human mammalian		
Non-human non-mammalian		

Category 5. Rate Publication Bias

Possible ratings: 0=no change; -1 or -2 downgrade 1 or 2 levels.

GRADE (Guyatt et al. 2011b) and Cochrane (Higgins and Green 2011) assess publication bias in a similar manner. Whereas “selective outcome reporting” is assessed for each study included in the review as part of the risk of bias assessment, “publication bias” is assessed on the body of evidence. GRADE states that “when an entire study remains unreported and the results relate to

the size of the effect- publication bias- one can assess the likelihood of publication bias only by looking at a group of studies."

Cochrane's definition of publication bias is "the *publication* or *non-publication* of research findings depending on the nature and direction of the results." Cochrane and GRADE are primarily concerned with *overestimates* of true effects of treatments or pharmaceuticals, especially related to "small studies effects", i.e., the tendency for estimates of an intervention to be more beneficial in smaller studies. There is empirical evidence in the clinical sciences that publication and other reporting biases result in over estimating the effects of interventions (Higgins and Green 2011).

In contrast, with the Navigation Guide, we are primarily concerned with *underestimating* the true effects of a chemical exposure, since in many cases population wide exposure has already occurred. Applying this inverted concern to GRADE's assessment for publication bias, leads to these considerations when rating publication bias:

- Early *negative* studies, particularly if small in size, are suspect. (GRADE is concerned with early *positive* studies).
- Authors of systematic reviews should suspect publication bias when studies are uniformly small, particularly when sponsored by the industry. (Same as GRADE)
- Empirical examination of patterns of results (e.g., funnel plots) may suggest publication bias but should be interpreted with caution. (Same as GRADE)
- More compelling than any of these theoretical exercises is authors' success in obtaining the results of some unpublished studies and demonstrating that the published and unpublished data show different results. (Same as GRADE)
- Comprehensive searches of the literature including unpublished studies, i.e., the grey literature, and a search for research in other languages are important to addressing publication bias. Note that Cochrane also states "comprehensive searching is not sufficient to prevent some substantial potential biases.

**Type of study**	**Publication bias rating**	**Rationale for your judgment**
Human		
Non-human mammalian		
Non-human non-mammalian		

Category 6. Rate Factors that Can Increase Quality of Evidence

Possible ratings: 0=no change; +1 or +2 upgrade 1 or 2 levels.

GRADE states that the circumstances for upgrading likely occur infrequently and are primarily relevant to observational and other non-randomized studies. Although it is possible to rate up results from randomized controlled trials, GRADE has yet to find a compelling circumstance for doing so (Guyatt et al. 2011e).

GRADE specifies 3 categories for increasing the quality of evidence (Guyatt et al. 2011e):

1. Large magnitude of effect. Modeling studies suggests that confounding (from non-random allocation) alone is unlikely to explain associations with a relative risk (RR) greater than 2 (or less than 0.5), and very unlikely to explain associations with an RR greater than 5 (or less than 0.2). Thus, these are the definitions of "large magnitude of effect" used to upgrade 1 or 2 levels, respectively. Also, GRADE is more likely to rate up if the effect is rapid and out of keeping with prior trajectory; usually supported by indirect evidence. GRADE presents empirical evidence to support these conclusions, and states that "although further research is warranted, both modeling and empirical work suggest the size of bias from confounding is unpredictable in direction but bounded in size. Hence, the GRADE group has previously suggested guidelines for rating quality of evidence up by one category (typically from low to moderate) for associations greater than 2, and up by two categories for associations greater than 5."

2. Dose-response gradient. Possible considerations include consistent dose response gradients in one or multiple studies, and/or dose response across studies, depending on the overall relevance to the body of evidence.

3. All plausible residual confounders or biases would reduce a demonstrated effect, or suggest a spurious effect when results show no effect. GRADE provides the following example of grading up evidence when observational studies have failed to demonstrate an association. Observational studies failed to confirm an association between vaccination and autism. This lack of association occurred despite the empirically confirmed bias that parents of autistic children diagnosed after the publicity associated with the article that originally suggested this relationship would be more likely to remember their vaccine experience than parents of children diagnosed before the publicity and presumably, than parents of non-autistic children. The negative findings despite this form of recall bias suggest rating up the quality of evidence.

Type of study	Large magnitude of effect rating	Rationale for your judgment
Human		

The results of the reviewers' ratings by population will be compiled and discussed leading to a final decision on overall quality of human evidence. The rationale for the decision will be fully documented.

1. **Final decision on overall quality of human evidence:**

(Example: Moderate quality is upgraded 1 step to high for Xyz reason(s))

---- High

---- Moderate

---- Low

---- Very

2. **Final decision on overall quality of non-human mammalian evidence:**

(Example: High quality is downgraded 1 step to moderate for Xyz reason(s))

---- High

---- Moderate

---- Low

---- Very

3. **Final decision on overall quality of non-human non-mammalian evidence:**

(Example: High quality is downgraded 1 step to moderate for Xyz reason(s))

---- High

---- Moderate

---- Low

---- Very

II. Rate the Strength of Evidence

The evidence quality ratings will be translated into strength of evidence for each population based on a combination of four criteria: (1) Quality of body of evidence; (2) Direction of effect; (3) Confidence in effect; and (4) Other compelling attributes of the data that may influence certainty (Figures 2 and 3). These strength of evidence ratings are linked to Tables 1 and 2, below, where their meaning is defined.

III. Combine Strength of Evidence For Human and Non-human Evidence

The final step in the process is to combine the strength of the evidence according to the chart in Figure 1. Combining the strength of evidence for human and non-human data will be produce an

overall statement of toxicity, i.e., known to be toxic to fetal growth; probably toxic to fetal growth; possibly toxic to fetal growth; known to be not-toxic to fetal growth.

List of studies included in systematic review of the relationship between birth weight and maternal glomerular filtration rate

Observational human studies—fetal growth and glomerular filtration rate

1) Akahori Y, Masuyama H, Hiramatsu Y. 2012. The correlation of maternal uric acid concentration with small-for-gestational-age fetuses in normotensive pregnant women. Gynecol Obstet Invest 73(2): 162-167.
2) Davison JM, Hytten FE. 1974. Glomerular filtration during and after pregnancy. J Obstet Gynaecol Br Commonw 81(8): 588-595.
3) Dunlop W, Furness C, Hill LM. 1978. Maternal haemoglobin concentration, haematocrit and renal handling of urate in pregnancies ending in the births of small-for-dates infants. Br J Obstet Gynaecol 85(12): 938-940.
4) Duvekot JJ, Cheriex EC, Pieters FA, Menheere PP, Schouten HJ, Peeters LL. 1995. Maternal volume homeostasis in early pregnancy in relation to fetal growth restriction. Obstet Gynecol 85(3): 361-367.
5) Faupel-Badger JM, Hsieh CC, Troisi R, Lagiou P, Potischman N. 2007. Plasma volume expansion in pregnancy: implications for biomarkers in population studies. Cancer Epidemiol Biomarkers Prev 16(9): 1720-1723.
6) Gibson HM. 1973. Plasma volume and glomerular filtration rate in pregnancy and their relation to differences in fetal growth. J Obstet Gynaecol Br Commonw 80(12): 1067-1074.
7) Knopp RH, Bergelin RO, Wahl PW, Walden CE. 1985. Relationships of infant birth size to maternal lipoproteins, apoproteins, fuels, hormones, clinical chemistries, and body weight at 36 weeks gestation. Diabetes 34 Suppl 2: 71-77.
8) Laughon SK, Catov J, Roberts JM. 2009. Uric acid concentrations are associated with insulin resistance and birthweight in normotensive pregnant women. Am J Obstet Gynecol 201(6): 582 e581-586.

Observational human studies—fetal growth and plasma volume expansion

9) Bernstein IM, Wulfkuhle K, Schonberg A. 2010. Fetal growth restriction is associated with reduced maternal plasma volume expansion. Abstract. Reproductive Sciences 1): 103A.
10) Blankson ML, Goldenberg RL, Cutter G, Cliver SP. 1993. The Relationship between Maternal Hematocrit and Pregnancy Outcome - Black-White Differences. Journal of the National Medical Association 85(2): 130-134.
11) Boomer AL, Christensen BL. 1982. Antepartum hematocrit, maternal smoking and birth weight. J Reprod Med 27(7): 385-388.

12) Dunlop W, Furness C, Hill LM. 1978. Maternal haemoglobin concentration, haematocrit and renal handling of urate in pregnancies ending in the births of small-for-dates infants. Br J Obstet Gynaecol 85(12): 938-940.
13) Gallery EDM, Saunders DM, Hunyor SN, Gyory AZ. 1979. Relationship between plasma-volume expansion and intra-uterine fetal growth in normal and hypertensive pregnancy. Abstract. Aust N Z J Obstet Gynaecol 19(3): 179-179.
14) Gibson HM. 1973. Plasma volume and glomerular filtration rate in pregnancy and their relation to differences in fetal growth. J Obstet Gynaecol Br Commonw 80(12): 1067-1074.
15) Hays PM, Cruikshank DP, Dunn LJ. 1985. Plasma volume determination in normal and preeclamptic pregnancies. Am J Obstet Gynecol 151(7): 958-966.
16) Hutchins CJ. 1980. Plasma volume changes in pregnancy in Indian and European primigravidae. Br J Obstet Gynaecol 87(7): 586-589.
17) Hytten FE, Stewart AM, Palmer JH. 1963. The relation of maternal heart size, blood volume and stature to the birth weight of the baby. J Obstet Gynaecol Br Commonw 70: 817-820.
18) Hytten FE, Paintin DB. 1963. Increase in plasma volume during normal pregnancy. J Obstet Gynaecol Br Emp 70: 402-407.
19) Keet MP, Jaroszewicz AM, van Schalkwyk DJ, Deale CJ, Odendaal HJ, Malan C, et al. 1981. Small-for-age babies: etiological factors in the Cape colored population. S Afr Med J 60(5): 199-203.
20) Lu ZM, Goldenberg RL, Cliver SP, Cutter G, Blankson M. 1991. The Relationship between Maternal Hematocrit and Pregnancy Outcome. Obstetrics and Gynecology 77(2): 190-194.
21) Pirani BB, Campbell DM, MacGillivray I. 1973. Plasma volume in normal first pregnancy. J Obstet Gynaecol Br Commonw 80(10): 884-887.
22) Rajalakshmi K, Raman L. 1985. Plasma volume changes in Indian women with normal pregnancy. Indian J Med Res 82: 521-527.
23) Rosso P, Donoso E, Braun S, Espinoza R, Fernandez C, Salas SP. 1993. Maternal hemodynamic adjustments in idiopathic fetal growth retardation. Gynecol Obstet Invest 35(3): 162-165.
24) Sagen N, Nilsen ST, Kim HC, Bergsjo P, Koller O. 1984. Maternal hemo globin concentration is closely related to birth weight in normal pregnancies. Acta Obstetricia et Gynecologica Scandinavica 63(3): 245-248.
25) Salas SP, Rosso P, Espinoza R, Robert JA, Valdes G, Donoso E. 1993. Maternal plasma-volume expansion and hormonal changes in women with idiopathic fetal growth-retardation. Obstetrics and Gynecology 81(6): 1029-1033.
26) Salas SP, Rosso P. 1998. Plasma volume, renal function, and hormonal levels in pregnant women with idiopathic fetal growth restriction or preeclampsia. Hypertension in Pregnancy 17(1): 69-79.
27) Scanlon KS, Yip R, Schieve LA, Cogswell ME. 2000. High and low hemoglobin levels during pregnancy: differential risks for preterm birth and small for gestational age. Obstet Gynecol 96(5 Pt 1): 741-748.

28) Steer P, Alam MA, Wadsworth J, Welch A. 1995. Relation between Maternal Hemoglobin Concentration and Birth-Weight in Different Ethnic-Groups. Br Med J 310(6978): 489-491.
29) Ueland K. 1976. Maternal cardiovascular dynamics. VII. Intrapartum blood volume changes. Am J Obstet Gynecol 126(6): 671-677.
30) von Tempelhoff GF, Heilmann L, Rudig L, Pollow K, Hommel G, Koscielny J. 2008. Mean maternal second-trimester hemoglobin concentration and outcome of pregnancy: a population-based study. Clin Appl Thromb Hemost 14(1): 19-28.
31) Whittaker PG, Macphail S, Lind T. 1996. Serial hematologic changes and pregnancy outcome. Obstet Gynecol 88(1): 33-39.
32) Gibson HM. 1973. Plasma volume and glomerular filtration rate in pregnancy and their relation to differences in fetal growth. J Obstet Gynaecol Br Commonw 80(12): 1067-1074.
33) Varga I, Rigo J, Jr., Somos P, Joo JG, Nagy B. 2000. Analysis of maternal circulation and renal function in physiologic pregnancies; parallel examinations of the changes in the cardiac output and the glomerular filtration rate. J Matern Fetal Med 9(2): 97-104.

Observational non-human mammalian studies—fetal growth and glomerular filtration rate

34) Abeni F, Bergoglio G, Masoero G, Terzano GM, Allegrini S. 2004. Plasma hormones and metabolites in Piedmontese cows during late pregnancy: relationships with calf birth weight. J Anim Sci 82(2): 438-444.

Observational non-human mammalian studies—fetal growth and plasma volume expansion

35) Haynes DM. 1954. Uterine blood volume. Obstet and Gynecol 3((5)): 517-522.

Experimental non-human mammalian studies—fetal growth and plasma volume expansion

36) Rumball CW, Bloomfield FH, Harding JE. 2008. Cardiovascular adaptations to pregnancy in sheep and effects of periconceptional undernutrition. Placenta 29(1): 89-94.
37) Van Mieghem T, van Bree R, Van Herck E, Deprest J, Verhaeghe J. 2009. Insulin-like growth factor-II regulates maternal hemodynamic adaptation to pregnancy in rats. Am J Physiol Regul Integr Comp Physiol 297(5): R1615-1621.

References

Balshem H, Helfand M, Schunemann HJ, Oxman AD, Kunz R, Brozek J, et al. 2011. GRADE guidelines: 3. Rating the quality of evidence. J Clin Epidemiol 64(4): 401-406.

Guyatt G, Oxman A, Kunz R, Brozek J, Alonso-Coello P, Rind D, et al. 2011. GRADE guidelines: 6. Rating the quality of evidence--imprecision. J Clin Epidemiol 64(12): 1283-1293.

Guyatt GH, Oxman AD, Akl EA, Kunz R, Vist G, Brozek J, et al. 2011a. GRADE guidelines: 1. Introduction-GRADE evidence profiles and summary of findings tables. J Clin Epidemiol 64(4): 383-394.

Guyatt GH, Oxman AD, Montori V, Vist G, Kunz R, Brozek J, et al. 2011b. GRADE guidelines: 5. Rating the quality of evidence-publication bias. J Clin Epidemiol.

Guyatt GH, Oxman AD, Kunz R, Woodcock J, Brozek J, Helfand M, et al. 2011c. GRADE guidelines: 8. Rating the quality of evidence-indirectness. J Clin Epidemiol.

Guyatt GH, Oxman AD, Kunz R, Woodcock J, Brozek J, Helfand M, et al. 2011d. GRADE guidelines: 7. Rating the quality of evidence-inconsistency. J Clin Epidemiol.

Guyatt GH, Oxman AD, Sultan S, Glasziou P, Akl EA, Alonso-Coello P, et al. 2011e. GRADE guidelines: 9. Rating up the quality of evidence. J Clin Epidemiol.

Halvorson GC. 2008. Electronic medical records and the prospect of real time evidence development. In: Evidence-based medicine and the changing nature of health care: 2007 IOM annual meeting summary. Washington, DC:The National Academies Press.

Hemminki K, Vineis P. 1985. Extrapolation of the evidence on teratogenicity of chemicals between humans and experimental animals: chemicals other than drugs. Teratog Carcinog Mutagen 5(4): 251-318.

Higgins JPT, Green S. 2011. Cochrane Handbook for Systematic Reviews of Interventions Version 5.1.0 The Cochrane Collaboration.

Institute of Medicine, Eden J, Wheatley B, McNeil B, Sox H. 2008. Knowing what works in health care: a roadmap for the nation: National Academies Press.

Kimmel CA, Holson JF, Hogue CJ, Carlo G. 1984. Reliability of Experimental Studies for Predicting Hazards to Human Development. (NCTR Technical Report for Experiment No 6015). Jefferson, AR.

National Research Council (U.S.) Committee on Developmental Toxicology, National Research Council (U.S.) Commission on Life Sciences. 2000. Scientific frontiers in developmental toxicology and risk assessment. Washington, DC: National Academy Press.

Nemec MD, Kaufman LE, Stump DG, Lindstrom P, Varsho BJ, Holson JF. 2006. Significance, Reliability, and Interpretation of Developmental and Reproductive Toxicity Study Findings. In: Developmental Reproductive Toxicology: A Practical Approach:Informa Healthcare.

Newman LM, Johnson EM, Staples RE. 1993. Assessment of the Effectiveness of Animal Developmental Toxicity Testing for Human Safety. Reprod Toxicol 7(4): 359-390.

Nisbet ICT, Karch NJ. 1983. Chemical Hazards to Human Reproduction. Park Ridge, NJ: Noyes Data Corp.

Peterson ED. 2008. Research methods to speed the development of better evidence- the registries example. In: Evidence-based medicine and the changing nature of health care:2007 IOM annual meeting summary. Washington, DC:The National Academies Press.

Viswanathan M, Ansari M, Berkman N, Chang S, Hartling L, McPheeters L, et al. 2012. Assessing the Risk of Bias of Individual Studies in Systematic Reviews of Health Care Interventions. (Agency for Healthcare Research and Quality Methods Guide for Comparative Effectiveness Reviews). AHRQ Publication No. 12-EHC047-EF.
